# Not all neuroligin 3 and 4X missense variants lead to significant functional inactivation

**DOI:** 10.1002/brb3.793

**Published:** 2017-08-14

**Authors:** Xiaojuan Xu, Zhengmao Hu, Lusi Zhang, Hongfang Liu, Yuemei Cheng, Kun Xia, Xuehong Zhang

**Affiliations:** ^1^ The Reproductive Medicine Hospital of the First Hospital of Lanzhou University Lanzhou Gansu China; ^2^ The Key Laboratory for Reproductive Medicine and Embryo Lanzhou Gansu China; ^3^ The State Key Laboratory of Medical Genetics and School of Life Science Central South University Changsha Hunan China; ^4^ Department of Ophthalmology Second Xiangya Hospital Central South University Changsha Hunan China; ^5^ Second School of Clinical Medicine of Lanzhou University Lanzhou Gansu China

**Keywords:** autism, functional inactivation, neuroligin, postsynaptic cell adhesion, variation

## Abstract

**Introduction:**

Neuroligins are postsynaptic cell adhesion molecules that interact with neurexins to regulate the fine balance between excitation and inhibition of synapses. Recently, accumulating evidence, involving mutation analysis, cellular assays, and mouse models, has suggested that neuroligin (NLGN) mutations affect synapse maturation and function. Previously, four missense variations [p.G426S (NLGN3), p.G84R (NLGN4X), p.Q162K (NLGN4X), and p.A283T (NLGN4X)] in four different unrelated patients have been identified by PCR and direct sequencing.

**Methods:**

In this study, we analyzed the functional effect of these missense variations by in vitro experiment via the stable HEK293 cells expressing wild‐type and mutant neuroligin.

**Results:**

We found that the four mutations did not significantly impair the expression of neuroligin 3 and neuroligin 4X, and also did not measurably inhibit the neurexin 1–neuroligin interaction. These variants might play a modest role in the pathogenesis of autism or might simply be unreported infrequent polymorphisms.

**Conclusion:**

Our data suggest that these four previously described neuroligin mutations are not primary risk factors for autism.

## 1 INTRODUCTION

Neuroligins are postsynaptic cell adhesion molecules that are involved in the NRXN‐NLGN‐SHANK pathway, most likely associated with synaptogenesis and the balance between synapse excitation and inhibition (Bang & Owczarek, 2013; Mackowiak, Mordalska, & Wedzony, 2014). Accumulating evidence suggests involvement of the neuroligin family in autism and other neuropsychiatric disorders (Jamain et al., 2003; Laumonnier et al., 2004; Lawson‐Yuen, Saldivar, Sommer, & Picker, 2008; Philippe et al., 1999; Sindi, Tannenberg, & Dodd, 2014; Thomas et al., 1999). In 2003, a mutation in neuroligin 3 (*NLGN3*; p.R451C) and a mutation in neuroligin 4X (*NLGN4X*; p.D396 fs) were reported in two unrelated Swedish autism families (Jamain et al., 2003). Since then, several other mutations in the *NLGN3*,* NLGN4X*, and *NLGN4Y* genes have been reported to be related to autism (Chih, Afridi, Clark, & Scheiffele, 2004; Daoud et al., 2009; Kuroda et al., 2014; Laumonnier et al., 2004; Lawson‐Yuen et al., 2008; Talebizadeh et al., 2006; Yan et al., 2005, 2008). In vitro and in vivo experiments have indicated that the autism‐related neuroligin mutations may affect synapse maturation and function.(Bemben et al., 2015; Chih et al., 2004; Ey, Leblond, & Bourgeron, 2011; Jaramillo, Liu, Pettersen, Birnbaum, & Powell, 2014; Zhang et al., 2009).

In our previous study, we identified one *NLGN3* and three *NLGN4X* variants (p.G426S in *NLGN3*; p.G84R, p.Q162K, and p.A283T in *NLGN4X*) (Xu et al., 2014). All four were missense and located in the conserved extracellular noncatalytic acetylcholinesterase homology domain, which is essential for binding to neurexin and triggering synaptic activity. Prediction of the effect of these substitutions using MutationTaster (http://www.mutationtaster.org/), Poly‐Phen‐2 (http://genetics.bwh.harvard.edu/pph2/), and SIFT (http://sift.jcvi.org/) revealed that p.G84R and p.A283T were “probably damaging”; however, p.G426S and p.Q162K were likely to be “benign” (Xu et al., 2014). The four variants might impair the functional properties of neuroligin related to synaptic homeostasis, thereby increasing predisposition to autism. To test this hypothesis, here we analyzed the functional effect of these four missense variants.

## 2 MATERIAL AND METHODS

### 2.1 Plasmids and mutagenesis

Vectors expressing unmodified human neuroligin 3 (NM_018977.2) and neuroligin 4X (NM_020742.2) were subcloned into a green fluorescent protein (GFP)‐tagged (C terminal, pEGFP‐N1) vector, and verified by sequencing. Vectors expressing a soluble IgG‐NX1β fusion protein (in a pCMV5 backbone) were provided by Professor Thomas C. Südhof (Zhang et al., 2009).

The four human *NLGN3* or *NLGN4X* mutation [p.G426S (*NLGN3*), p.G84R (*NLGN4X*), p.Q162K (*NLGN4X*), and p.A283T (*NLGN4X*)] plasmids and two reported positive‐control human *NLGN3* and *NLGN4X* mutation [p.R451C (*NLGN3*) and p.R87W (*NLGN4X*)] plasmids were constructed by introducing the relevant point mutation using a Agilent Native Pfu QuickChange Kit (Agilent, US, Santa Clara, California).

The molecular weight for all of the GFP‐NL3 fusion proteins is ≈110 kDa(including the wild‐type protein, mutant protein, and the positive‐control). The molecular weight for the GFP‐NL4‐WT and the three GFP‐NL4‐mutant fusion proteins is ≈120 kDa. The molecular weight for the GFP‐NL4‐ R87W fusion proteins is ≈90 kDa. The molecular weight for the loading control beta‐actin is ≈42 kDa.

### 2.2 Cell culture and transfections

Transfection experiments used HEK293 cells with Lipofectamine^®^ 2000 Transfection Reagent (Invitrogen, US, Carlsbad, California). Before transfection, the medium was replaced with Opti‐MEM^™^, then 0.8 μg plasmid, 100 μl Opti‐MEM^™^, and 2 μl Lipofectamine 2000 (Invitrogen) was used for each well of a 24‐well plate. After 4–6 hr, the medium was replaced with Dulbecco's modified Eagle's medium (DMED)(Hyclone, USA, Logan, Utah). Stably transfected cells were selected by growth in G418 (Geneticin, 800–1200 μg/ml; Sigma, US, Santa Clara, California) ‐contained DMED Feeder and sustained by growth in G418 (200 μg/ml) contained Feeder.

### 2.3 Assay of mutant protein degradation rate

Stable HEK293 cell lines expressing wild‐type or mutant neuroligin 3 were pretreated with 20 μg/ml cycloheximide (CHX, Sigma, US, Santa Clara, California) for 0 (untreated), 3, 6, 9, or 12 hr. Cells expressing wild‐type or mutant neuroligin 4X were pretreated with 20 μg/ml CHX for 0 (untreated), 4, 8,12, 16, or 24 hr. After the indicated time the culture medium was removed and the cells washed twice with 1× phosphate‐buffered saline (PBS). Then 25 μl 2× sodium dodecyl sulfate (SDS) sample buffer (with protease inhibitor) was added, plus boil it ready for Western blotting. Beta‐actin was used as a loading control. Western‐blot gray scale of triplicate experiments was performed by the software Image J (Clontech Laboratories, Inc. Cat# 632381 RRID:AB_2313808; Jackson ImmunoResearch Labs Cat# 115‐005‐062 RRID:AB_2338452).

### 2.4 Assay of mutant protein degradation pathway

Stable HEK293 cell lines expressing wild‐type or mutant neuroligin were pretreated with the proteasome inhibitor MG132 (10 μmol/L, Sigma, US, Santa Clara, California) or the lysosome inhibitor chloroquine (CQ; 100 μmol/L; Sigma, US, Santa Clara, California) for 0 or 10 hr. After the indicated time the culture medium was removed and the cells washed twice with 1× PBS. Then 25 μl 2× SDS sample buffer (with protease inhibitor) was added, plus boil it ready for Western blotting. Beta‐actin was used as a loading control. Western blot gray scale of triplicate experiments was performed by the software Image J(Clontech Laboratories, Inc. Cat# 632381 RRID:AB_2313808; Jackson ImmunoResearch Labs Cat# 115‐005‐062 RRID:AB_2338452).

### 2.5 Immunofluorescence

Stable HEK293 cell lines were grown on coverglasses in a 24‐well plate. After 24 hr, the culture medium was removed and the cells washed twice with 1× PBS. The cells were then fixed with 4% paraformaldehyde/4% sucrose for 15 min, permeabilized with 0.2% Triton X‐100 in PBS for 10 min, and incubated with 5% bovine serum albumin in PBS for 30 min. The cells were then incubated with the indicated primary antibody (Sigma or Invitrogen) for 1 hr followed by AlexaFluor 488‐ or 546‐labeled goat anti‐mouse or anti‐rabbit antibody (Jackson ImmunoResearch) for 1 hr. For immunostaining, anti‐GFP (1:200, Thermo Fisher Scientific Cat# A‐11122 also A11122 RRID:AB_221569, US, Carlsbad, California),anti‐golgi (1:200; an Golgi body marker; BD Biosciences Cat# 611281 RRID:AB_398809, UK, Cambridge)and anti‐calnexin (1:200; an endoplasmic reticulum [ER] marker; BD Biosciences Cat# 610524 RRID:AB_397884, US, Franklin Lakes, New Jersey) antibodies were used. Images were captured using a Leica TCS SP5 confocal microscope.

### 2.6 Assay of cell surface biotinylation

Stable HEK293 cell lines were grown on coverglasses in 6‐cm plate. After 24 h, the culture medium was removed and the cells washed twice with 1× PBS. For surface biotinylations, the cells were washed twice with ice‐cold PBS, pH 8.0 and incubated with 2 mg/ml sulfo‐NHS‐LC‐biotin (Pierce) in PBS, pH 8.0 for 30 min on ice. in at 4°C) and the beads washed 3–5 times with 1× PBS (with protease inhibitors). Then, 100 μl Quenching Solutio (with protease inhibitors) was added to terminate reaction. Subsequently, cells were washed with 1× PBS (with protease inhibitors). The proteins were extracted for 10 min at 4°C in Lysis Buffer (PBS containing 1% Triton X‐100, 0.2% SDS, 5 mmol/L EDTA, 2 mmol/L DTT, and protease inhibitors), and the lysate saved in 1.5‐ml centrifuge tubes. Insoluble material was removed by centrifugation (1,000*g* for 5 min), and the supernatant was quantified by Pierce BCA protein assay and retained for the biotin labeling. The 100 μl NeutrAvidin Agarose (Thermo Fisher Scientific) were washed three times with 1 ml 1× PBS (with protease inhibitors) by centrifugation (1,000*g* for 3 min). Then part of the supernatant (including 500 μg protein) was mixed with the NeutrAvidin Agarose and rotated at 4°C for at least 6 hr. The supernatant was removed by centrifugation (1,000*g* for 4 min at 4°C) and the beads washed 3–5 times in 1× PBS (with protease inhibitors) by centrifugation (1,000*g* for 4 min at 4°C). Then bound proteins were eluted by boiling in 2× SDS sample buffer and analyzed by Western blotting. Ratios of surface to lysate pools of neuroligin proteins were estimated by densitometric scanning of blots derived from three independent experiments. Western blot gray scale of triplicate experiments was performed by the software Image J(Clontech Laboratories, Inc. Cat# 632381 RRID:AB_2313808; Jackson ImmunoResearch Labs Cat# 115‐005‐062 RRID:AB_2338452).

### 2.7 NX1 expression and purification

The rProtein A Sepharose Fastflow Kits (GE Healthcare) was used to purify soluble IgG‐NX1β fusion protein. HEK293 cells were transfected with plasmid expressing the IgG‐NX1β fusion protein, using Lipofectamine 2000. After 72 hr, the supernatant was collected, and protease inhibitors (pepststin,leupeptin,aprotinin and PMSF, a final concentrati of 0.1 g/L; Sigma, US, Santa Clara, California) plus 1 mol/L HEPES‐NaOH were added. Insoluble material was removed by centrifugation (2,500*g* for 20 min), and the supernatant was retained for the purification assay. Each binding assay used 10 μl IgG beads. The beads were washed three times with 1 ml 1× PBS (with protease inhibitors) by centrifugation (6,500*g* for 5 min). Then the supernatant was mixed with the beads and rotated at 4°C for at least 6 hr. The supernatant was removed by centrifugation (6,500*g* for 20 min at 4°C) and the beads washed 3–5 times in 1× PBS (with protease inhibitors). Then elution buffer (glycine, 100 mmol/L) was added and the tubes rotated at room temperature for 30 min. The IgG beads were removed by centrifugation (6,500*g* for 5 min at 4°C). Neutralizing buffer (2 mol/L Tris‐HCl, pH 8.0) was added to the supernatant and it was saved for immunoprecipitation (IP). Western blot gray scale of triplicate experiments was performed by the software Image J (Clontech Laboratories, Inc. Cat# 632381 RRID:AB_2313808; Jackson ImmunoResearch Labs Cat# 109‐165‐088 RRID:AB_2337725; Jackson ImmunoResearch Labs Cat# 115‐005‐062 RRID:AB_2338452).

### 2.8 Neurexin 1–neuroligin interaction assay

The interaction between neurexin 1 and neuroligin was tested by IP. The soluble IgG‐NX1β fusion protein could be immunoprecipitated by anti‐IgG. Neuroligin could be detected by anti‐GFP. Stable HEK293 cell lines expressing wild‐type or mutant neuroligin (in a pEGFP‐N1 backbone) were washed with 1× PBS (with protease inhibitors). The proteins were extracted for 10 min at 4°C in Co‐IP buffer (10 mmol/L Hepes, 2.5 mmol/L CaCl_2_, 1% TritonX‐100, 10% glycerol and 150 mmol/L NaCl, plus protease inhibitors), and the lysate saved in 1.5‐ml centrifuge tubes. The supernatant, containing 2 mg neuroligin, was mixed with 50 μg IgG‐NX1β fusion protein and 10 μl beads, then rotated at 4°C for at least 6 hr. The supernatant was removed by centrifugation (6,500*g* for 20 min at 4°C) and the beads washed 3–5 times with 1× PBS (with protease inhibitors). Then, 25 μl 4× SDS sample buffer (with protease inhibitors) was added, plus Boil it ready for Western blotting to detect neuroligin using anti‐GFP. Western blot gray scale of triplicate experiments was performed by the software Image J (Clontech Laboratories, Inc. Cat# 632381 RRID:AB_2313808; Jackson ImmunoResearch Labs Cat# 109‐165‐088 RRID:AB_2337725; Jackson ImmunoResearch Labs Cat# 115‐005‐062 RRID:AB_2338452).

### 2.9 Statistical analysis

In this study, all statistical analysis was performed using Prism 5 software (GraphPad). The two tailed Student's t‐test was used for the comparison of the two groups. The two‐way ANOVA and Dunnett's multiple comparison test was used for the comparison between the three groups or more than three groups. *p* values lower than .05 were considered to be significant.

## 3 RESULTS

To study the functional effect of the four mutations, we analyzed the expression level, subcellular localization, degradation, and interaction with neurexin, using in vitro experiments in HEK293 cells stably expressing wild‐type or mutant neuroligin 3 or neuroligin 4X.

**Figure 1:**
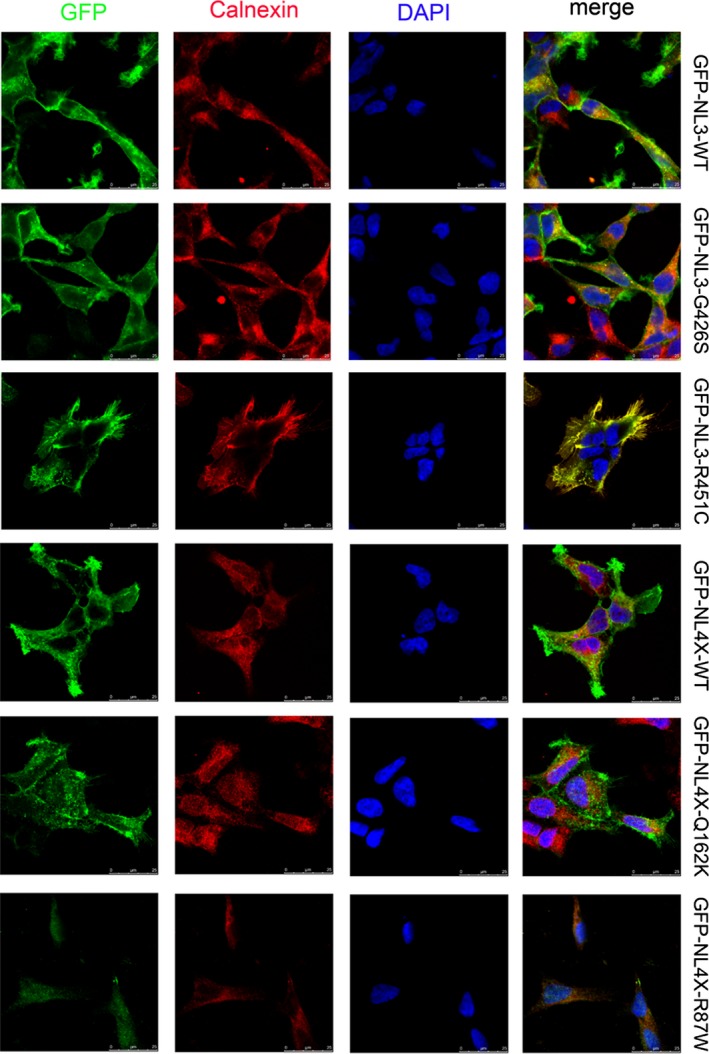
The two variants did not block the transport of neuroligin from ER to cell membrane. HEK293 cells expressing GFP‐tagged wild‐type neuroligin (NL3‐WT and NL4‐WT), mutant neuroligin (NL3‐G426S and NL4‐Q162K), and positive control (NL3‐R451C and NL4‐R87W) were immunostained with antibodies against the GFP tag (first column, green) and against the ER‐marker calnexin (second column, red). The nucleus was labeled by DAPI (4′,6‐diamidino‐2‐phenylindole) (third column, blue). Both the wild‐type and mutant neuroligin are transported efficiently to the cell surface, whereas the positive control accumulates in the ER, as confirmed by the overlap with the ER‐resident protein calnexin. The scale bar is 5 mm

**Figure 2:**
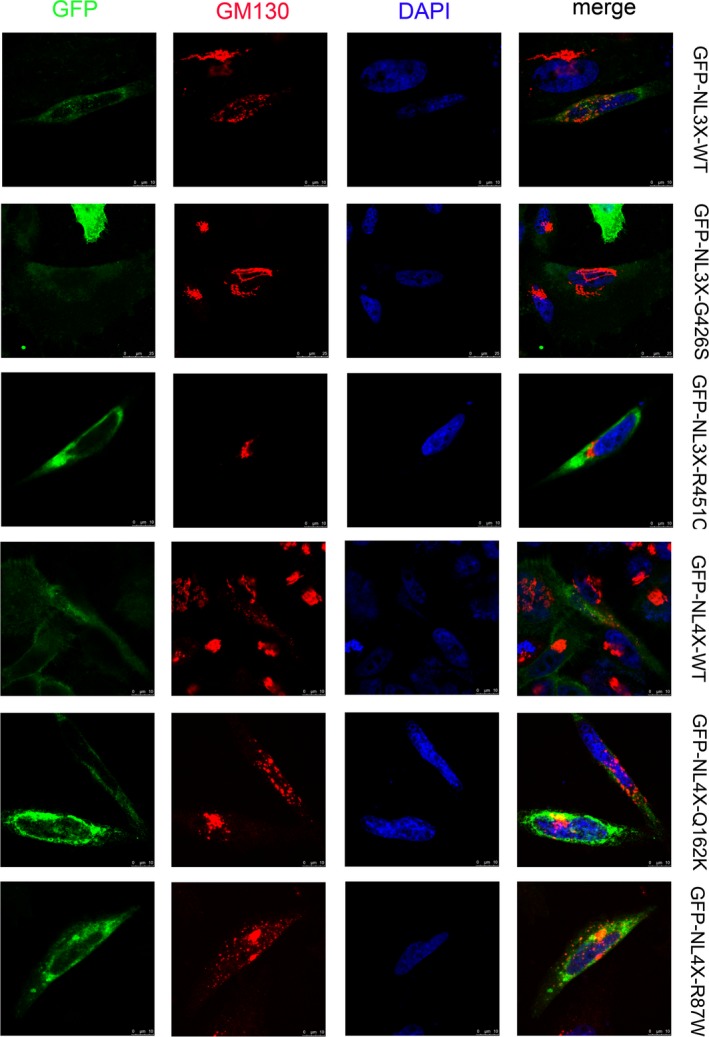
The two variants did not block the transport of neuroligin from ER to cell membrane. HEK293 cells expressing GFP‐tagged wild‐type neuroligin (NL3‐WT and NL4‐WT), mutant neuroligin (NL3‐G426S and NL4‐Q162K), and positive control (NL3‐R451C and NL4‐R87W) were immunostained with antibodies against the GFP tag (first column, green) and against the Golgi‐marker GM130 (second column, red). The nucleus was labeled by DAPI (4′,6‐diamidino‐2‐phenylindole) (third column, blue). Both the wild‐type and mutant neuroligin are transported efficiently to the cell surface, whereas the positive control accumulates in the Golgi body, as confirmed by the overlap with the Golgi body‐resident protein calnexin. The scale bar is 5 mm

**Figure 3:**
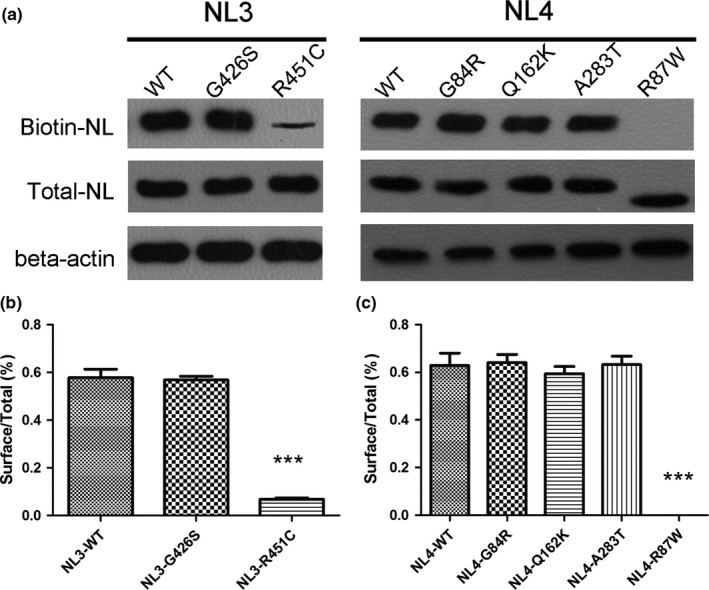
HEK293 cells expressing GFP‐tagged wild‐type neuroligin (NL3‐WT and NL4‐WT), mutant neuroligin (NL3‐G426S, NL4‐G84R, NL4‐Q162K, NL4‐A283T) and positive control (NL3‐R451C and NL4‐R87W) were analyzed for cell surface localization. Proteins were detected in total cell lysates by western blotting with anti‐GFP antibodies. Cell surface proteins were modified with membrane non‐permeable reactive biotin and modified proteins were isolated on NeutrAvidin Agarose. NLs in the biotinylated fraction (surface) were analyzed by western blotting with anti‐GFP antibodies. (a) Total cell protein and Cell surface protein expression of wild‐type and mutants NL3 and NL4 in the HEK293 cells. (b, c) Quantification for the percentage of cell surface protein expression to the total cell protein. *** referred to *p*<.0001

Immunofluorescence showed that mutant neuroligin 3 and neuroligin 4X did not colocalized with calnexin and GM130. In contrast, the two positive control mutant proteins colocalized with calnexin but not colocalized with GM130, indicating that they were mostly retained in the ER. Cell surface biotinylation indicated that mutant neuroligin 3 and neuroligin 4X were abundant in the cell surface fractions. However, we recovered few amount of NL3‐R451C(≈5%) and no NL4 R87W in the surface fraction. These demonstrated that mutant neuroligin 3 and neuroligin 4X were transported to the cell surface with similar efficiency as the wild‐type proteins (Figure 1, Figure 2, and Figure 3) (Data for NL4‐G84R and NL4‐A283T were not shown).

**Figure 4:**
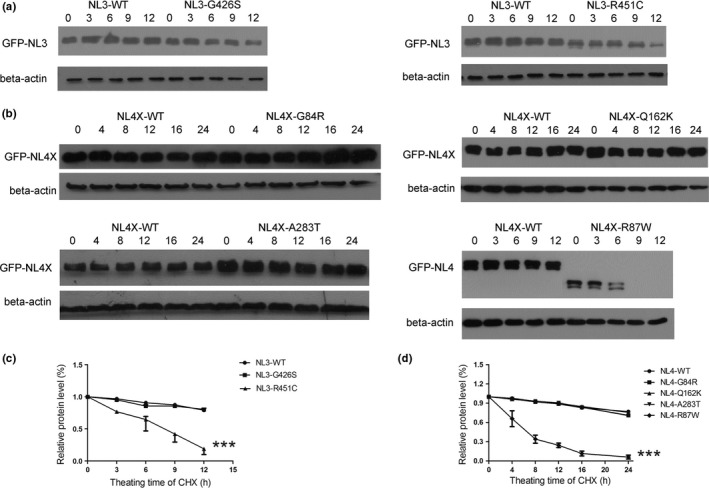
The four variants do not destabilize neuroligin 3 and neuroligin 4X. HEK293 cells expressing GFP‐tagged wild‐type neuroligin (NL3‐WT and NL4‐WT), mutant neuroligin (NL3‐G426S, NL4‐G84R, NL4‐Q162K, NL4‐A283T), and positive control (NL3‐R451C and NL4‐R87W) were treated with 20 μg/ml CHX for 0 to 12 hr (for NL3) or 0 to 24 hr (for NL4X). Inhibition of protein synthesis, and the accumulation of wild‐type neuroligin, mutant neuroligin, or positive control were evaluated by Western blotting. Beta‐actin was used as a loading control. (a) Degradation rate assay of mutant neuroligin 3. (b) Degradation rate assay of mutant neuroligin 4X. (c, d) Quantification for the assay of mutant protein degradation rate. *** referred to *p* < .0001

Cycloheximide (CHX) stops protein translation, allowing us to monitor the degradation rate of the mutant proteins. In the presence of CHX, the expression level of the four mutant proteins remained similar to that of wild‐type neuroligin 3 and neuroligin 4X (*p* > .05), whereas the two positive controls showed a rapid loss (*p* < .0001) (Figure 4). Thus, the four mutations did not destabilize neuroligin 3 or neuroligin 4X.

**Figure 5:**
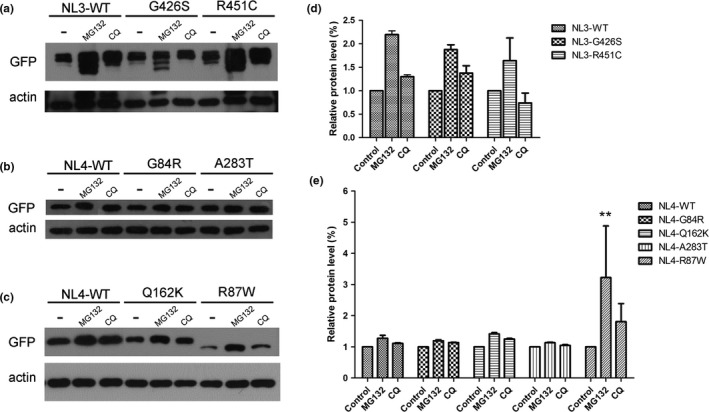
The four variants do not change the degradation pathway of neuroligin 3 and neuroligin 4X. HEK293 cells expressing GFP‐tagged wild‐type neuroligin (NL3 and NL4X), mutant neuroligin (G426S, G84R, Q162K, and A283T), or positive control (R451C and R87W) were pretreated with the proteasome inhibitor MG132 (10 μmol/L) or the lysosome inhibitor chloroquine (CQ; 100 μmol/L) for 0 hr (untreated) or 10 hr. The accumulation of wild‐type neuroligin, mutant neuroligin, and positive control was evaluated by Western blotting. Beta‐actin was used as a loading control. (a) Degradation pathway assay of mutant neuroligin 3. (b, c) Degradation pathway assay of mutant neuroligin 4X. (d, e) Quantification for the assay of mutant protein degradation pathway. ** referred to *p* < .001

The proteasome inhibitor MG132 selectively inhibits protein degradation by the ubiquitin‐proteasome. The lysosome inhibitor CQ selectively inhibits protein degradation by autophagy. In the presence of inhibitor, we observed an clear increase in mutant neuroligin 3 and neuroligin 4X—the same as for their wild‐type equivalents (*p* > .05) (Figure 5). The two positive control mutants showed the same clear increase with MG132 treatment and a mild increase with CQ treatment (*p* < .001). This suggests that the four mutations and the two positive controls do not change the protein degradation pathways of neuroligin 3 and neuroligin 4X.

**Figure 6:**
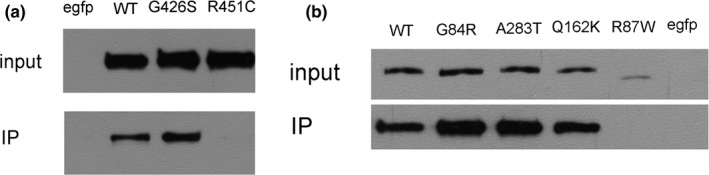
The four variants do not impair the interaction of neuroligin 3 and neuroligin 4X with neurexin 1. The neurexin 1–neuroligin interaction was measured by IP. The soluble IgG‐NX1β fusion protein could be immunoprecipitated by anti‐IgG. Neuroligin could be detected by anti‐GFP. Inputs: lysates of HEK293 cells expressing GFP‐tagged wild‐type neuroligin, mutant neuroligin, or positive control. IP: lysates after IP assay. (a) Assay for mutant neuroligin 3. (b) Assay for mutant neuroligin 4X

Because the four mutant proteins did not impair the folding of neuroligin 3 and neuroligin 4X and also did not inhibit their surface export, we measured the ability of these mutant proteins to bind to recombinant IgG‐NX1β fusion protein. We observed that the mutant neuroligin 3 and neuroligin 4X showed a binding affinity for their cognate partner, β‐neurexin, that was similar to that of wild‐type neuroligin (Figure 6). Therefore, neurexin‐binding was not impaired by any of the four mutations. However, the two positive controls did not bind to recombinant IgG‐NX1β fusion protein.

## 4 DISCUSSION

Autism is a complex neurodevelopmental disorder in which mutations in neuroligin may play a role. In this study, we used in vitro assays in HEK293 cells to demonstrate that four identified neuroligin mutations did not affect protein expression or interaction with neurexin 1. We conclude that these four variants either play only a modest role in predisposition to autism, or are previously unreported infrequent polymorphisms rather than pathogenic mutations. We must bear in mind, however, that we cannot recapitulate the situation in a human patient: we were studying a single factor in isolation, ignoring other genetic, epigenetic, and environmental factors that may play a contributory role in disease causation.

In conclusion, our data indicate that our four previously described mutations in neuroligins 3 and 4X are not primary risk factors for autism.

## CONFLICT OF INTEREST

The authors have no disclosures to declare.
